# A new assessment tool to measure the socio-economic impact of food allergy on patients and parents: the Food Allergy Socio-Economic Questionnaire Short-Form (FASEQ-SF)

**DOI:** 10.1186/2045-7022-5-S3-P3

**Published:** 2015-03-30

**Authors:** Eimear Morrissey, Donal Moynihan, Kasia Pyrz, Jonathan O B Hourihane, Audrey Dunn Galvin

**Affiliations:** 1 University College Cork, Cork, Ireland

## Aim

Food Allergy may have an impact on socioeconomic (SE) aspects of life. The Europrevall study (EU FP6, 2005-2009) developed and validated a SE questionnaire to measure the direct, indirect and intangible costs of food allergy. This measure takes up to 1 hour to complete as participants are required to respond to 70 to 170 questions, depending on response choice.

We aimed to develop a brief, reliable, standardized measure of the socio-economic impact of food allergy on patients' everyday lives.

## Methods

In Stage 1, data with responses from 134 parents was subjected to principal components analysis to investigate factor structure, including overlap and duplication, and to remove redundant items that do not contribute meaningfully to subscale and overall scale score. The results of this analysis gave rise to the prototype short form measure.

In Stage 2, 50 parents of children in Cork University Hospital, with children aged between 6 months and 13 years completed the new short measure. The data was subjected to psychometric analysis, including an assessment of scale and item reliability and construct validity.

## Results

The Short measure has 30 questions on indirect, direct, and socio-emotional costs and can be completed in 15 minutes. Analyses demonstrated high scale and inter-item reliability (mean α = 0.80). There were strong correlations (range 0.7 - 0.9) between subscales evaluating socio-emotional costs, direct costs, indirect costs, severity of symptoms, and frequency of reactions.

## Conclusion

The FASEQ-SF provides a quick, easy reliable alternative to a long questionnaire that will accurately capture the socio-economic impact of food allergy on patients lives. It will ensure quality of measurement while reducing the cost and effort of participants and researchers. It is presently being validated in a cohort of adult patients.

